# Dramatic Response of Lupus Enteritis, Nephritis, and Pancytopenia to Plasmapheresis and Rituximab

**DOI:** 10.1155/2022/3443141

**Published:** 2022-06-06

**Authors:** Adan Aftab, Nida Saleem, Syed Farhat Abbas, Zafar Ullah, Muhammad Haneef

**Affiliations:** Shifa International Hospital, H-8/4, Pitras Bukhari Road, Islamabad, Pakistan

## Abstract

**Background:**

Although lupus enteritis is a rare manifestation of systemic lupus erythematosus yet results in significant distress. This disorder contributes to diagnostic and therapeutic dilemma leading to enhanced mortality. *Case Description*. We report a case history of a 29-year-old female who presented with severe abdominal pain, watery stools, and vomiting, and later on, she developed pancytopenia and renal impairment. On intensive workup, diagnosis of lupus-associated enteritis, nephritis, and pancytopenia was discovered. She improved drastically on initiation of plasmapheresis followed by low-dose intravenous rituximab. One year posttreatment, she remained in complete remission.

**Conclusion:**

From this case, it can be suggested that in a young female with intractable abdominal pain, the remote possibility of lupus enteritis must be kept in mind. Besides this, plasmapheresis can have a potential role in refractory lupus enteritis. Furthermore, low-dose intravenous rituximab can be a safe and cost-effective treatment option in achieving sustained remission of clinical and laboratory parameters in lupus enteritis.

## 1. Introduction

Systemic lupus erythematosus (SLE) is a multisystem autoimmune disorder affecting predominantly young females. Many gastrointestinal manifestations can occur in SLE, which can be related to disease itself, superimposed infections, and gastrointestinal side effects of immunosuppressant given for its management. The reported incidence of these manifestations is between 1.3 and 27.5% [1]. Lupus enteritis is a rare manifestation of SLE with a reported prevalence of around 3.5–5.8% in Asian population [2], caused by formation of immune complexes against small vessels of the gut, predominantly the jejunum and ileum [3], leading to necrotizing vasculitis. The classical manifestation of this disorder is severe acute abdominal pain. Abdominal pain in SLE can be secondary to peritonitis, pancreatitis, acalculous cholecystitis, gastroenteritis, mesenteric ischemia, intestinal pseudoobstruction, and peptic ulcer disease [1, 3, 4], thus making diagnosis of this rare debilitating condition even more difficult. Lupus enteritis, if remained undiagnosed, can lead to gastrointestinal ischemia, infarction leading to bleeding, and perforation requiring emergent surgical intervention [1] and is associated with a high mortality rate. Therefore, deeper understanding of this disease entity is necessary. Several studies have mentioned the therapeutic role of several immunosuppressive medications in improving clinical and laboratory parameters. However, no study has described about the potential role of plasmapheresis and low-dose intravenous rituximab in achieving sustained remission. We report the first case of severe acute onset lupus enteritis with nephritis and pancytopenia responded immediately upon initiation of plasmapheresis with no recurrence after low-dose intravenous rituximab therapy.

## 2. Case Report

The patient, a 29-year-old female resident of Gujrat, Pakistan, married, with no known comorbidities, admitted to our hospital through emergency department with severe abdominal pain, loose stools, and vomiting for one week. These symptoms had not responded to the intravenous antibiotics and analgesics, which she received at some local hospital. Her symptoms worsened three days before admission. At that time, there was an increase in the severity of pain, and loose stools became more watery in consistency with mucus in them. However, there was no blood in stools. Besides this, she had unintentional weight loss of around seven kilograms with generalized weakness and joint pains for the last three months. Her family history was insignificant for any bowel or autoimmune disorder.

On examination, she was pale. Her blood pressure was 100/60 mmHg, pulse was 92/min, and she was afebrile. Her abdominal examination was significant for severe abdominal tenderness with hypoactive bowel sounds. Rest of the systemic examination was unremarkable. Postadmission, her workup was performed, as given in Table 1.

As given in Table 1, there is evidence of pancytopenia, raised serum creatinine, proteinuria, folic acid deficiency, hypokalemia, and presence of mucus with white blood cells on stool routine examination.

She was kept nil per oral due to ongoing diarrhea and vomiting. She received intravenous fluids for volume depletion. She also received intravenous meropenem and metronidazole along with syrup vancomycin for suspected infectious diarrhea. Also, intravenous tramadol and nalbuphine on as need basis was prescribed for abdominal pain relief. To control vomiting and dyspeptic symptoms, omeprazole infusion and intravenous ondansetron were administered. Intravenous folinic acid was given for correction of folic acid deficiency.

At this point, diagnosis of inflammatory bowel disease and mesenteric ischemia was suspected. In order to identify the exact cause of her symptoms, CT vascular angiography was performed which showed extensive mucosal enhancement, thickening, and marked submucosal edema in the rectum, sigmoid, and descending colon up to the splenic flexure. However, there was relative sparing of ascending and transverse colon. Moreover, there was mild hepatosplenomegaly and ascites. All the vessels were patent with no evidence of engorgement of mesenteric vasculature. These findings were suggestive of severe acute proctocolitis.

**Figure 1:**
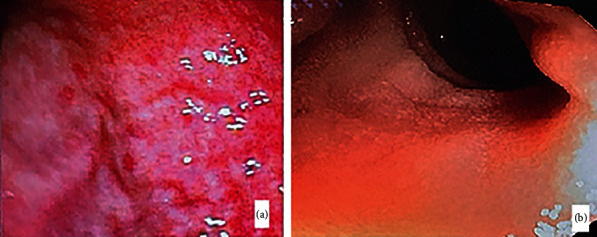
(a) Endoscopic image showing the evidence of hemorrhagic gastritis. (b) Colonoscopic view showing the presence of bowel wall edema.

Considering her history and radiological findings, upper GI endoscopy and colonoscopy were done as shown in Figure 1.

**Figure 2:**
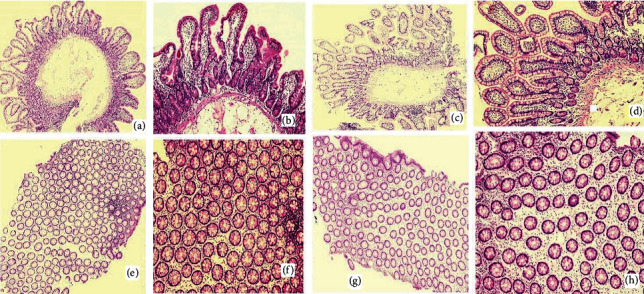
Light microscopic images (H&E staining). Normal duodenal lining: (a) (4X). (b) (10X). Normal ileal lining: (c) (4X). (d) (10X). Normal rectal lining: (e) (4X). (f) (10X). Normal lining of sigmoid colon: (g) (4X). (h) (10X).

**Figure 3:**
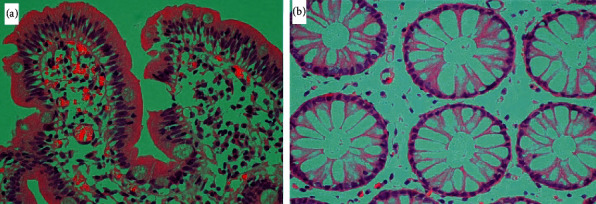
Light microscopic images (H&E staining). (a) Normal duodenal lining (40X). (b) Normal colonic lining (40X).

Multiple biopsies from different segments including the sigmoid colon, rectum, gastric body, and ileum were taken. Figures 2 and 3 show light microscopic appearance of different GI segments at different power fields.

As shown in above figures, there is no evidence of malignancy and granulomatous disease. Congo red staining was negative for amyloidosis as well.

**Figure 4:**
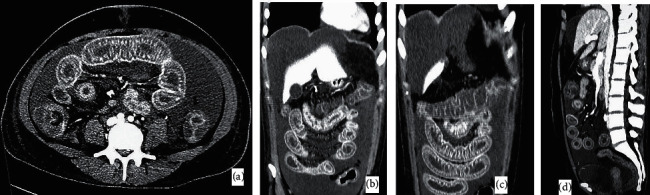
Markedly thickened edematous bowel loops at different segments of GI tract with extensive mucosal enlargement. Patent abdominopelvic vessels with no evidence of ischemia. (a) Axial view, (b)-(c) coronal view, and (d) sagittal view.

In addition to this, diagnostic laparoscopy was performed which showed moderate abdominopelvic ascites. Her peritoneal fluid cytology was negative for malignancy. Contrast enhanced CT scan abdomen (CECT) was repeated, which showed worsening of the disease process with panenteritis involving large and small bowel up to the esophagus.  Figure 4 shows CECT abdomen images.

As her condition deteriorated further, her autoimmune workup was done to identify any autoimmune pathology.

Results are given in Table 2.

As given in Table 2, there are low serum complement levels (c3, c4) and positive ANA, anti-Ro, and anti-La antibodies, indicating the possible diagnosis of systemic lupus erythematosus.

Keeping in view of the above, diagnosis of lupus enteritis was made. She was then started on intravenous methylprednisolone 250 mg once daily, for 3 days, which was then increased to 500 mg once daily, for 5 days after no symptomatic improvement. In spite of these interventions, no symptomatic relief was obtained and her abdominal pain persisted. In order to relieve her abdominal pain, pain management department was consulted and epidural bupivacaine infusion was started. Despite of opioids administration and even the neuromuscular blocking agent, her pain continued.

**Figure 5:**
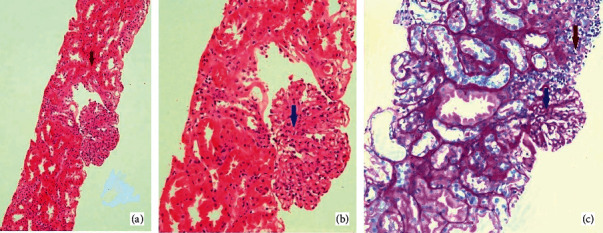
H&E stain at (a) 10X and (b) 40X. (c) PAS stain at 40X. Blue arrow represents the marked endocapillary hypercellularity and proliferation. Maroon arrow represents the tubulointerstitial inflammation.

Meanwhile, tablet mycophenolate mofetil (MMF) was initiated at a low dose (250 mg twice daily) due to low total leucocyte count. After 3 days, MMF was increased to 500 mg twice daily. However, no symptomatic relief was achieved. Later, after 4 days, plasmapheresis was initiated using albumin as a replacement fluid. She responded dramatically to 5 daily sessions of plasmapheresis with complete resolution of all her symptoms in the next few days, and later on, she was off the analgesics as well. As her serum creatinine was 3 mg/dL with ongoing proteinuria, therefore, her renal biopsy was performed.  Figure 5 shows histopathological images obtained after renal biopsy.

The above figure shows marked endocapillary hypercellularity consistent with diffuse proliferative glomerulonephritis (class IV lupus nephritis) and moderate tubular injury. Immunofluorescence showed granular IgG and c3 deposits in glomerular basement membrane and mesangium. Mesangial IgM and C1q containing immune deposits were focally positive as well. There was no evidence of tubulointerstitial fibrosis. She remained stable postplasmapheresis, her complete blood count normalized, serum creatinine decreased to around 2 mg/dL, and later on, she was discharged on mycophenolate mofetil 1000 mg twice daily, hydroxychloroquine (HCQ) 200 mg twice daily, and tapering doses of steroids.

On follow up, 7 days later, she again developed lupus-associated pancytopenia. As she was young female, in her reproductive age group, therefore, intravenous cyclophosphamide was not considered. She was successfully treated with 100 mg weekly doses of intravenous rituximab. After total 4 doses of rituximab, her total leucocyte count, hemoglobin, and platelet count normalized.

**Figure 6:**
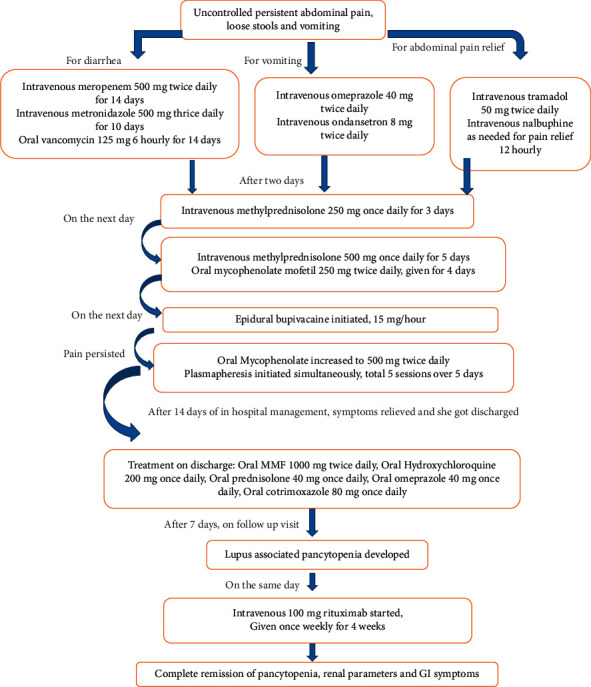
Summary of treatment received by the patient.

One year following 5 sessions of plasmapheresis and 4 doses of rituximab, steroids, MMF, and HCQ, she is in complete remission from all perspectives, which include settlement of abdominal pain and diarrhea, normalization of complete blood picture, and most importantly, renal parameters. Her current serum creatinine is 0.65 mg/dL and urine albumin to creatinine ratio is 33 mg/g. The flowchart in Figure 6 shows the treatment received by the patient.

## 3. Discussion

As previously reported [3–5], acute intense abdominal pain and vomiting is the predominant clinical feature of lupus enteritis. Our patient had similar clinical manifestations, which were not responding to antimicrobial medications and wide range of analgesics. Therefore, it can be concluded that in a young female with nonresponding abdominal pain and ongoing vomiting and diarrhea, the remote possibility of lupus-induced gastrointestinal (GI) vasculopathy must be kept in mind.

As abdominal pain in SLE can be due to multiple causes and there is no available confirmatory investigation that has high sensitivity and specificity [1], multiple diagnostic clues can aid in accurate diagnosis of lupus enteritis. These include the preexisting history of SLE with hypocomplementemia suggestive of active lupus, nonresponding abdominal pain, extensive bowel wall edema, mesenteric vascular engorgement and enhanced mesenteric fat attenuation on abdominal CT scan, nondiagnostic histopathological evidence, and endoscopic findings.

Few case studies have been published regarding degree of GI involvement in lupus-induced vasculopathy. According to our knowledge, this is the third case report describing about extensive involvement of the gastrointestinal tract from the esophagus to the rectum [5, 6].

There are several reported causes of pancytopenia in SLE patients. These include nutritional deficiency, autoimmune myelofibrosis [7], destruction of cell lines by circulating immune complexes [8], hypersplenism, and haemophagocytic lymphohistiocytosis [9]. Pancytopenia in our patient was likely secondary to immune complex-mediated destruction of blood cell lines as indicated by the positive Coombs test and superimposed folic acid deficiency. However, this cannot be said conclusively, as no bone marrow biopsy was done. Our patient responded immediately upon initiation of four weekly doses of 100 mg intravenous rituximab.

In SLE, several potential therapeutic roles of high-dose intravenous rituximab have been mentioned in the literature. These include role of rituximab in management of lupus-associated diffuse alveolar hemorrhages, lupus-induced retinal vasculitis [10], recurrent pancreatitis [11], refractory lupus nephritis [12], neuropsychiatric lupus, lupus-induced transverse myelitis [13], lupus-induced pulmonary hypertension [14], refractory cutaneous lupus [15], lupus-induced panniculitis [16], lupus myocarditis [17], and catastrophic antiphospholipid antibody (APLA) syndrome. Regarding lupus-induced hematological abnormalities, it has been well known that high-dose rituximab therapy can correct lupus-induced aplastic anemia [18], thrombotic thrombocytopenic purpura (TTP) [19], and refractory autoimmune hemolytic anemia [20].

However, regarding efficacy of low-dose (100 mg weekly, 4 doses) rituximab, very limited data are available. Only few studies have been conducted regarding the potential role of low-dose rituximab in SLE, and these are only about lupus-induced thrombocytopenia [21, 22]. As low-dose intravenous rituximab has played a role in inducing sustained remission of lupus-induced GI vasculitis and pancytopenia, as seen in our patient, therefore, it can be suggested that low-dose rituximab has added advantages of cost-effectiveness and limited side effect profile and can be utilized as an effective option for refractory systemic lupus and lupus-induced pancytopenia. However, this preposition needs further validation.

In several studies, the therapeutic role of plasmapheresis in SLE has been mentioned. The use of plasmapheresis has been proposed for the management of lupus-induced diffuse alveolar hemorrhages, retinal vasculitis [10], posterior reversible encephalopathy syndrome [23], refractory TTP [24], lupus cerebritis [25], and catastrophic APLA [26]. However, according to our knowledge, for refractory lupus-induced GI vasculitis, no role of plasmapheresis has been mentioned in the literature.

Several studies have mentioned improvement in clinical and laboratory parameters with steroids [3, 5], mycophenolate mofetil (MMF) [27], and cyclophosphamide [28] and have mentioned the potential role of tacrolimus [28] and rituximab for recurrent lupus enteritis [29]. Our patient had persistent abdominal pain in spite of high-dose steroids and MMF and even required opioid analgesics along with anesthetic medication. In one study, it has been suggested to undergo laparotomy for abdominal pain not responding to immunosuppressants [2]. However, after getting an insight about dramatic response to plasmapheresis in our patient, it can be proposed that plasmapheresis can be used as a last resort in lupus-induced gastrointestinal vasculopathy, refractory to conventional immunosuppressive therapy.

As mentioned, overall reported mortality of lupus enteritis was 53% [2]. The major contributing factors leading to the enhanced mortality rate are late diagnosis and initiation of immunosuppression. However, in Asian population, benign clinical course and excellent prognosis of this distressful disorder have been mentioned [30, 31]. Although the response to steroids and MMF has not been satisfactory in providing symptomatic relief to our patient, yet after commencement of plasmapheresis and later on low-dose rituximab, patient remained in complete remission even after one year of therapy.

Few studies have described about the risk factors which determine the likelihood of gastrointestinal recurrence, which includes preexisting positive antiphospholipid antibodies (APLA), bowel wall thickness of more than 9 mm on CT scan [2], low-dose or early steroid withdrawal [30], and even rituximab [29] and cyclophosphamide [31] free immunosuppression protocol, have been associated with early recurrence. Absence of APLA antibodies, administration of high-dose pulse steroids with gradual tapering, and subsequent rituximab administration reduced the possibility of recurrence of lupus enteritis, as seen in our patient. However, to validate these findings, further studies are needed.

It can be concluded that in a young female with SLE and intractable abdominal pain, the possibility of lupus-associated abdominal vasculopathy must be kept in mind. Second, it is of utmost importance to diagnose early and manage this condition urgently in order to avoid life-threatening complications and surgical intervention. Third, as seen from this case, plasmapheresis can play a significant role in immediate correction of clinical and laboratory parameters in immunosuppressant refractory cases, thus precluding the immediate need for surgical intervention. Finally, low-dose rituximab can be tried in cases of nonresponding lupus-associated pancytopenia and can provide sustained remission of lupus-associated gastrointestinal vasculopathy as well. However, further studies are needed to validate these salient findings.

## Figures and Tables

**Table 1:** Postadmission laboratory investigations.

Post-Admission Laboratory Investigations (Table 1):			
Total leucocyte count	2500/*µ*L	Serum amylase	57 U/L
Platelet count	112000/*µ*L	Serum protein electrophoresis	No paraproteins
Hemoglobin	10.4 g/dL	Serum creatinine	3 mg/dL
Reticulocyte count	0.9%	BUN^e^	40 mg/dL
Transferrin saturation	37%	Sodium	143 meq/L
Vitamin B12	256 pm/L	Potassium	3.1 meq/L
Folic acid	<3.1 ng/mL	Chloride	112 meq/L
LDH^j^	238 U/L	Bicarbonate	20 meq/L
Coombs direct	Positive	Ionized calcium	4.3 mg/dL
Peripheral film	1% schistocyte	C-reactive protein	9.45 mg/dL
AST^a^	38 U/L	ESR^f^	11 mm/h
ALT^b^	12 U/L	Urine R/E^d^	3+ protein, 1+ blood
Total bilirubin	0.59 mg/dL	Albumin to creatinine ratio	1.343 g/g
GGT^c^	13 U/L	Stool R/E^d^	Mucus ++, 8–10 WBCs^i^
Alkaline phosphatase	37 U/L	Stool for *Clostridium difficile* (PCR^h^)	Negative
Serum lipase	17 U/L	Stool and blood C/S^g^	Negative

^a^Aspartate aminotransferase, ^b^alanine aminotransferase, ^c^gamma glutamyl transferase, ^d^routine examination, ^e^blood urea nitrogen, ^f^erythrocyte sedimentation rate, ^g^culture and sensitivity, ^h^polymerase chain reaction, ^i^white blood cells, ^j^lactate dehydrogenase.

**Table 2:** Autoimmune workup.

Autoimmune workup (Table 2):			
c3^a^	0.43 G/L ↓	Lupus anticoagulant	Negative
c4	0.15 G/L ↓	Anticardiolipin IgG, IgM	Negative
p-ANCA^b^	Negative	Anti-ds^f^DNA antibody	Negative
c-ANCA^c^	Negative	ANA^d^	2 positive nucleolar patterns
Anti-Ro	87 ↑	Antihistone antibody	Negative
Anti-La	61↑	Anti-TTG^e^ IgA antibody	Negative
Fecal calprotectin	Negative	Antiendomysial antibody	Negative

^a^Complement-3, ^b^perinuclear antineutrophilic cytoplasmic antibodies, ^c^circulating antineutrophilic cytoplasmic antibodies, ^d^antinuclear antibody, ^e^tissue transglutaminase antibody, ^f^double stranded.

## Data Availability

The data used to support this study are included within the article.
